# Nearly Complete Genome Sequence of Raoultella ornithinolytica Strain MQB_Silv_108, Carrying an Uncommon Extended-Spectrum-β-Lactamase-like *bla*_BEL_ Gene

**DOI:** 10.1128/mra.01012-22

**Published:** 2022-10-31

**Authors:** Marcos Quintela-Baluja, Kelly Jobling, David W. Graham, Mariano Gomez-Lopez, Jesus L. Romalde, Jorge Barros-Velázquez, Pilar Calo-Mata

**Affiliations:** a Department of Analytical Chemistry, Nutrition, and Food Science, School of Veterinary Sciences, University of Santiago de Compostela, Lugo, Spain; b School of Engineering, Newcastle University, Newcastle upon Tyne, United Kingdom; c UDE Engineering Ltd., Newcastle upon Tyne, United Kingdom; d Department of Microbiology and Parasitology, Center for Biological Research, Faculty of Biology, Universidade de Santiago de Compostela, Santiago de Compostela, Spain; e CRETUS, Universidade de Santiago de Compostela, Santiago de Compostela, Spain; University of Southern California

## Abstract

Raoultella ornithinolytica has become increasingly important in human diseases. Here, we report the nearly complete genome sequence of a multidrug-resistant strain, R. ornithinolytica MQB_Silv_108, which was isolated from the effluent from a domestic wastewater treatment plant in Spain. Therefore, its release into the environment poses a possible exposure risk for humans and animals.

## ANNOUNCEMENT

*Raoultella* species are aerobic, Gram-negative, nonmotile, encapsulated bacilli belonging to the *Enterobacteriaceae* family (1). *Raoultella* spp. occur in aquatic and soil environments and have traditionally been considered a rare cause of human infections (2). However, Raoultella ornithinolytica has become increasingly important in human diseases (3, 4), with infections being underreported because it is difficult to identify by routine biochemical and phenotypic methods (3, 5). In addition, R. ornithinolytica causes histamine poisoning in fish (scombroid syndrome) and, after consumption, also in humans, due to its capacity to convert histidine to histamine (6, 7).

Here, we report the complete genome of the multidrug-resistant R. ornithinolytica strain MQB_Silv_108, which was isolated in a ChromID extended-spectrum β-lactamase (ESBL) medium (bioMérieux, France) at 37°C overnight from the effluent from a wastewater treatment plant in a city in northwest Spain. One bacterial colony was picked and streaked on a new ESBL plate (twice) to obtain a pure isolate. The isolate was identified using a matrix-assisted laser desorption ionization (MALDI) Biotyper system (Bruker Daltonik, Bremen, Germany) according to the manufacturer’s instructions. The closest match was to R. ornithinolytica (score of 2.33).

Strain MQB_Silv_108 was grown from stocks in LB agar (Anachem, UK) and incubated at 37°C for 16 h. For Illumina sequencing, the bacterial biomass was harvested, resuspended with Microbank beads (Pro-Lab Diagnostics, UK), and shipped to MicrobesNG (Birmingham, UK) for genomic DNA extraction and sequencing using a 250-bp paired-end protocol (https://microbesng.com/documents/5/MicrobesNG_Methods_Document_-_PDF.pdf). Biomass from the same inocula was used for Oxford Nanopore Technologies (ONT) sequencing, using the QIAamp DNA minikit (Qiagen, Germany) for genomic DNA extraction.

Libraries for Illumina sequencing were prepared using the Nextera XT library preparation kit (Illumina, San Diego, CA, USA), while the ONT library was prepared without size selection using the SQK-LSK108 kit (ONT, UK) and sequenced using an R9.4 flow cell (FLO-MIN106; ONT). Both libraries were prepared following the kit manufacturer’s instructions. For ONT reads, base calling was performed using Guppy v4.0.11 with configuration dna_r9.4.1_450bps_hac (8), which yielded a total of 1,124,787 reads with an *N*_50_ value of 2,963 bp. The base-called reads were filtered with Filtlong v0.2.1 (9), using the Illumina reads as an external reference for quality assessment. A total of 1,291,110 Illumina reads were generated and subjected to quality control using fastp v023.2 (10).

The Illumina and ONT reads were used to construct a hybrid assembly with Unicycler v0.5.0 (11). The quality of the hybrid assembly was evaluated using QUAST v5.2.0 (12), and the genome completeness was assessed using Bandage v0.8.1 (13). The NCBI Prokaryotic Genome Annotation Pipeline (PGAP) v5.3 was used for genome annotation (14). The resistome was analyzed using ResFinder v4.1 (15), which identified genes conferring resistance to β-lactams, aminoglycosides, fluoroquinolones, phenicols, sulfonamides, trimethoprim, fosfomycin, and disinfectants. The plasmid replicons were identified with PlasmidFinder v2.1 (16). Sequencing metrics for the MQB_Silv_108 genome are summarized in Table 1. All tools were run with default parameters unless otherwise specified.

**TABLE 1:** Sequencing metrics for Raoultella ornithinolytica MQB_silv_108

Name	Genomic element	Sequence length (bp)	G+C content (%)	Avg coverage (×)^*a*^	Incompatibility group^*b*^	GenBank accession no.
MQB_Silv_108chr	Chromosome	5,775,339	55.3	122	NA	CP104450
pIncA_Silv108	Plasmid	148,888	52.6	434	IncA	CP104451
pIncFIB_Silv108^*c*^	Plasmid	138,718	51.4	328	IncFIB(K)	CP104452
pIncFII_Silv108	Plasmid	128,787	53.9	519	IncFII(pKP91)	CP104453
pMQB_Silv108.1	Plasmid	44,010	49.2	731	NI	CP104454
pIncP6_Silv108	Plasmid	22,326	58.9	1,738	IncP6	CP104455
pMQB_Silv108.2	Plasmid	13,466	52.6	1,577	NI	CP104456
pCol_Silv108.1	Plasmid	9,838	51.0	4,577	Col(pHAD28)	CP104457
pCol_Silv108.2	Plasmid	8,282	56.6	1,165	ColRNAI	CP104458
pMQB_Silv108.3	Plasmid	5,065	51.9	1,764	NI	CP104459
pMQB_Silv108.4	Plasmid	4,665	51.1	1,034	NI	CP104460
pMQB_Silv108.5	Plasmid	2,886	33.3	1,327	NI	CP104461

a The commands used for the quality filtering were as follows: for fastp, fastp –i read1.fastq –I read2.fastq –o filtered_read1.fastq –O filtered_read2.fastq; for Filtlong, filtlong −1 filtered_read1.fastq −2 filtered_read2.fastq –min_length 4000 –keep_percent 90 –trim –split 100 –mean_q_weight 10 basecalled.fastq.gz > filtered_long_read.fastq.

b NA, not applicable; NI, not identified using PlasmidFinder v2.0.

c Incomplete plasmid.

The nearly complete genome has a total size of 6,306,885 bp and consists of one circular chromosome, 10 complete circularized plasmids, and 1 incomplete plasmid. A total of 6,075 coding DNA sequences (CDSs) were annotated using PGAP v5.3. The *bla*_BEL_ gene was inserted in a new anthropogenic class 1 integron cassette array (*bla*_BEL-1_-*bla*_BEL-1_-*dfrB6*-*cmlA1v*) in a IncP6 plasmid (pIncP6_Silv108).

### Data availability.

The raw sequencing data are available in the NCBI Sequence Read Archive (SRA) under BioProject accession number PRJNA877288 with SRA accession numbers SRX17455753 (ONT) and SRX17455752 (Illumina). The complete genome and plasmid sequences have been deposited in GenBank under the accession numbers described in Table 1.
